# Pharmacological rescue of trafficking-impaired ATP-sensitive potassium channels

**DOI:** 10.3389/fphys.2013.00386

**Published:** 2013-12-24

**Authors:** Gregory M. Martin, Pei-Chun Chen, Prasanna Devaraneni, Show-Ling Shyng

**Affiliations:** Department of Biochemistry and Molecular Biology, Oregon Health & Science UniversityPortland, OR, USA

**Keywords:** ATP-sensitive potassium channel, pharmacological chaperone, sulfonylurea, carbamazepine, congenital hyperinsulinism (CHI)

## Abstract

ATP-sensitive potassium (K_ATP_) channels link cell metabolism to membrane excitability and are involved in a wide range of physiological processes including hormone secretion, control of vascular tone, and protection of cardiac and neuronal cells against ischemic injuries. In pancreatic β-cells, K_ATP_ channels play a key role in glucose-stimulated insulin secretion, and gain or loss of channel function results in neonatal diabetes or congenital hyperinsulinism, respectively. The β-cell K_ATP_ channel is formed by co-assembly of four Kir6.2 inwardly rectifying potassium channel subunits encoded by *KCNJ11* and four sulfonylurea receptor 1 subunits encoded by *ABCC8*. Many mutations in *ABCC8* or *KCNJ11* cause loss of channel function, thus, congenital hyperinsulinism by hampering channel biogenesis and hence trafficking to the cell surface. The trafficking defects caused by a subset of these mutations can be corrected by sulfonylureas, K_ATP_ channel antagonists that have long been used to treat type 2 diabetes. More recently, carbamazepine, an anticonvulsant that is thought to target primarily voltage-gated sodium channels has been shown to correct K_ATP_ channel trafficking defects. This article reviews studies to date aimed at understanding the mechanisms by which mutations impair channel biogenesis and trafficking and the mechanisms by which pharmacological ligands overcome channel trafficking defects. Insight into channel structure-function relationships and therapeutic implications from these studies are discussed.

## Introduction

ATP-sensitive potassium channels (K_ATP_) are a unique class of ion channels expressed in a variety of tissues including the pancreas, various regions of the brain, and cardiac, skeletal, and vascular smooth muscle (Aguilar-Bryan et al., 1998). By regulating K^+^ flux at the plasma membrane, they function as molecular sensors that couple cell metabolism to changes in membrane excitability (Nichols, 2006). The K_ATP_ channel is a hetero-octamer formed by a complex of two distinct protein subunits in 1:1 stoichiometry: an inwardly rectifying K^+^ channel Kir6.1/6.2 and a regulatory sulfonylurea receptor SUR1 or SUR2 (Inagaki et al., 1995, 1997; Clement et al., 1997; Shyng and Nichols, 1997). The “classic” channel subtype is composed of a tetramer Kir6.2, which forms the K^+^-conducting pore, with four surrounding SUR1 molecules, which provide regulatory functions.

**Figure 1:**
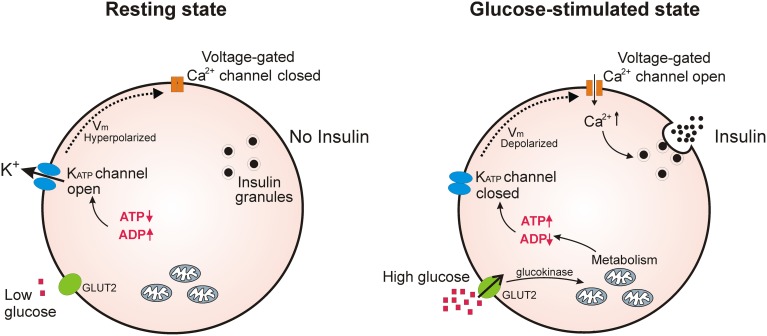
**K_ATP_ channels regulate insulin secretion in pancreatic β-cells**. Under conditions of low blood glucose, the relatively low ATP/ADP ratio in the β-cell promotes opening of K_ATP_ channels, keeping β-cell membrane potential at a hyperpolarized state to prevent Ca^2+^ influx and insulin release. Upon an increase in blood glucose, β-cells increase glucose uptake through GLUT2 transporters; glycolysis and respiration then elevate the intracellular ATP/ADP ratio and close K_ATP_ channels. This causes depolarization of the plasma membrane potential which opens voltage-gated Ca^2+^ channels; the subsequent influx of Ca^2+^ initiates fusion of insulin secretory granules with the plasma membrane.

In pancreatic β-cells, where K_ATP_ channels are best studied, they act as a key link in glucose-induced insulin secretion (Aguilar-Bryan and Bryan, 1999; Ashcroft, 2005). In these cells, fluctuations in the [ATP]/[ADP] ratio, brought about by changes in blood glucose levels, push the equilibrium of K_ATP_ channels toward the closed or open state. Thus, when blood glucose levels rise, the intracellular [ATP]/[ADP] ratio also rises, blocking K^+^ efflux through K_ATP_ channels. This depolarizes the β-cell and opens voltage-gated Ca^2+^ channels; the subsequent Ca^2+^ influx then triggers exocytosis of insulin secretory granules. When blood glucose levels fall, the intracellular [ATP]/[ADP] ratio will fall, pushing the equilibrium toward open K_ATP_ channels, repolarizing the β-cell and blocking further insulin release (Figure 1).

Not surprisingly, mutations in the genes encoding K_ATP_ channel subunits (*ABCC8* for SUR1 and *KCNJ11* for Kir6.2) often lead to a breakdown in glucose homeostasis. In general, mutations in K_ATP_ genes are classified as either gain-of-function, where constitutively open channels preclude insulin secretion, or loss-of-function, non-functional channels that are unable to hyperpolarize the β-cell and cause persistent insulin release (Aguilar-Bryan and Bryan, 1999; Hattersley and Ashcroft, 2005). Over the past 15 years, a number of groups have identified a class of loss-of-function mutations in the genes encoding the K_ATP_ channel, particularly in *ABCC8* (SUR1), that interfere with proper biogenesis and trafficking, thus, preventing normal surface expression of the channel (Cartier et al., 2001; Partridge et al., 2001; Taschenberger et al., 2002; Crane and Aguilar-Bryan, 2004; Tornovsky et al., 2004; Yan et al., 2004, 2007; Taneja et al., 2009). These mutations are collectively referred to as trafficking mutations. Studies have demonstrated that congenital hyperinsulinism of infancy (CHI), a rare disease characterized by persistent insulin secretion even under severe hypoglycemia (Stanley, 1997), is frequently caused by trafficking mutations in K_ATP_ channel genes. In these patients, channel subunits are synthesized but fail to reach the plasma membrane, mostly due to a disruption in the folding or oligomeric assembly process. The result is a constitutively depolarized β-cell with unregulated levels of insulin release. In many cases, the current therapy for these patients relies on partial or subtotal pancreatectomy to avoid permanent consequences of chronic hypoglycemia, which could lead to life-long insulin dependency.

Protein misfolding and mistrafficking resulting from genetic mutations underlie many human diseases. A prominent example is the ΔF508-CFTR (cystic fibrosis transmembrane conductance regulator) deletion mutation (Riordan et al., 1989), which is present in the majority of cystic fibrosis (CF) patients and causes defective folding, thereby inhibiting trafficking of the protein to the plasma membrane (Cheng et al., 1990). Small-molecule correctors, termed pharmacological chaperones, which specifically bind to a protein and enable its proper folding and localization, have been shown to correct trafficking defects in multiple disease-causing proteins, like ΔF508-CFTR (Hanrahan et al., 2013). In some cases, mutant proteins rescued to the correct cellular locations exhibit full or partial function to reverse disease phenotypes (Powers et al., 2009). Recent work has demonstrated that pharmacological chaperones may also hold promise in correcting trafficking-impaired K_ATP_ channels, offering new hope in the treatment of CHI.

In this review, we will discuss progress to date in defining the mechanisms by which mutations impair the biogenesis and trafficking of K_ATP_ channels and how these trafficking defects can be overcome using pharmacological approaches. In particular, we will describe the challenges facing pharmacological rescue of trafficking-impaired ion channels, and discuss the promises this area holds in the treatment of disease.

## Molecular composition of K_ATP_ channels

**Figure 2:**
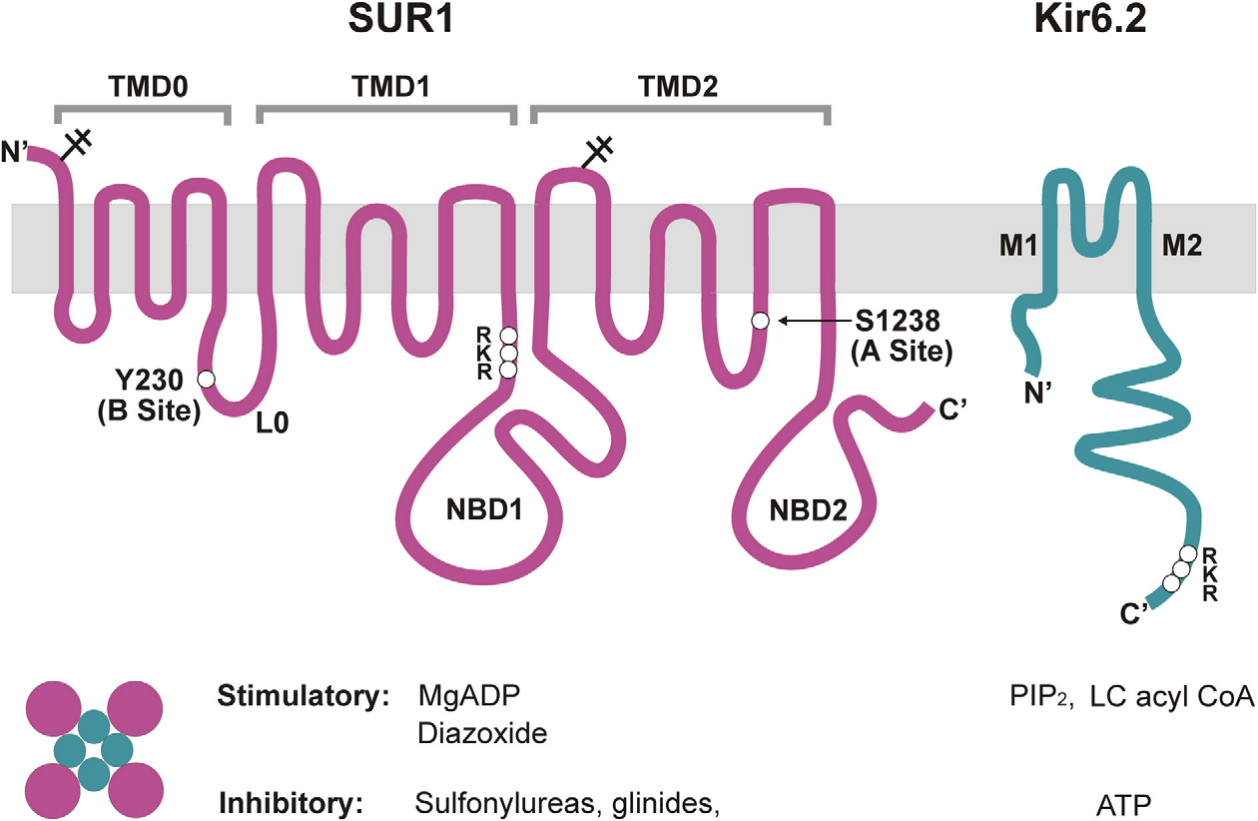
**Molecular composition and regulation of K_ATP_ channels**. Pancreatic K_ATP_ channels are hetero-octamers of four Kir6.2 subunits, which form the K^+^ conducting pore, and four regulatory SUR1 subunits. Shown on the top are transmembrane topologies of the two subunits. SUR1 has three transmembrane domains, TMD0, TMD1, and TMD2, two cytoplasmic nucleotide binding domains, NBD1 and NBD2, and a cytoplasmic linker L0 that connects TMD0 to the ABC core structure of the protein. Kir6.2 has two transmembrane helices, M1 and M2, and cytoplasmic N- and C-terminus. Physiological and pharmacological ligands that impact channel function are shown below. Mg-nucleotides interact with NBDs of SUR1 to activate the channel, whereas ATP binding at Kir6.2 closes the channel. PIP_2_ and LC acyl CoAs also interact with Kir6.2 but stimulate channel activity. Sulfonylureas and glinides inhibit, whereas diazoxide stimulates channel activity by interacting with primarily SUR1. For detailed discussion on the involvement of each subunit in channel regulation by the various physiological and pharmacological ligands please refer to the main text. Locations of the RKR motifs in SUR1 and Kir6.2 are marked. Y230 and S1238, SUR1 residues critical for site B and site A of the glibenclamide binding pocket are also marked.

The K_ATP_ channel is a large hetero-octamer of nearly 950 kDa, composed of four Kir6.2 and four SUR1 subunits (Clement et al., 1997) (Figure 2). A low-resolution cryo-EM structure indicates a compact configuration, 18 nm across and 13 nm in height, with a central tetrameric Kir6.2 core which forms the K^+^-conducting pore, embraced by four SUR1 proteins (Mikhailov et al., 2005).

Kir6.2 is a member of the potassium inward rectifier family (Kir). These channels have a greater tendency to allow ion flow into, rather than out of the cell owing to block by intracellular polyamines and Mg^2+^ at positive membrane potentials (Lopatin et al., 1994). In the case of weak inward rectifiers such as Kir6.2, the extent of intracellular block is less pronounced due to lack of strong binding sites for the positively charged blockers. Under most physiological conditions where membrane potential is positive to the equilibrium potential of K^+^ (E_K_), Kir channels generate small outward currents to keep membrane potential near the E_K_. This makes Kir channels primary regulators of resting membrane potential in cells that express them. Importantly, many Kir channels are ligand-gated, endowing them the ability to couple specific physiological signals to membrane excitability (Nichols and Lopatin, 1997). For the Kir6.2-SUR1 K_ATP_ channel complex, gating regulation by intracellular nucleotides ATP and ADP underlies its physiological function of coupling cell metabolism to cell excitability.

No definitive high resolution structure for Kir6 channels yet exists. However, homology modeling using crystal structures of eukaryotic and prokaryotic Kir channels has provided the basis for a structural model (Capener et al., 2000; Loussouarn et al., 2001). Thus, four Kir6.2 subunits combine to form the K^+^ pore. Each subunit has two transmembrane domains, M1 and M2, with intracellular N- and C-termini. Lying perpendicular to M1 and M2 is the short amphipathic “slide helix,” which links M1 to the short N-terminal domain. Mutagenesis studies have implicated this region in channel gating (Proks et al., 2004; Lin et al., 2006b; Li et al., 2013). Interspersing M1 and M2 is an extracellular linker followed by a pore-forming loop, constituting the selectivity filter, the region responsible for K^+^ ion specificity. The M2 helix connects to the large intracellular C-domain. This region constitutes nearly half of the protein, and mediates extensive interactions with adjacent Kir6.2 subunits (Antcliff et al., 2005).

SUR1 is a member of the ATP Binding Cassette (ABC)-C family of transporters, and is closely related to CFTR and MRP (multi-drug resistance related proteins). Homology modeling has been challenging without a solved structure for an ABC protein with significant sequence identity. Nonetheless, biochemical, electrophysiological, and mutagenesis studies have provided the essential topology and domain organization (Aguilar-Bryan et al., 1995; Tusnady et al., 1997; Conti et al., 2001) (Figure 2). As an ABC transporter, SUR1 has the core ABC structure of two transmembrane domains (TMD1 and TMD2), each consisting of 6 transmembrane helices. Each TMD is linked to a cytoplasmic nucleotide-binding domain (NBD1 and NBD2) by intracellular coupling domains (ICDs), α-helical extensions of the TMDs. Additionally, SUR proteins contain an N-terminal TMD0 domain of five transmembrane helices, plus a long cytoplasmic loop L0, linking TMD0 with the core ABC structure. TMD0 is absent from most ABC transporters, including CFTR, and seems to play a unique and interesting role in K_ATP_ channel biology. Studies implicate TMD0 in mediating interactions between SUR1 and Kir6.2 and modulating forward trafficking and gating of the channel (Schwappach et al., 2000; Babenko and Bryan, 2003; Chan et al., 2003). Another interesting point regarding SUR1 is the fact that this ABC-C transporter has no known function as a transporter; its role is strictly regulatory with regard to Kir6.2, at least in the systems examined so far. How these two proteins evolved to form a functional channel complex is a fascinating question.

## Biogenesis and trafficking regulation of K_ATP_ channels

Biogenesis and assembly of K_ATP_ channel proteins occurs in the ER (Zerangue et al., 1999). Not much is known regarding the events that couple translation of K_ATP_ channel subunits to insertion in the ER membrane however, or regarding details of the assembly process and molecular chaperones involved. When either SUR1 or Kir6.2 is expressed alone in heterologous systems, the subunits cannot escape the ER (Zerangue et al., 1999) and are presumably cleared through ER-associated degradation (ERAD), suggesting that assembled complexes are required for forward trafficking and surface expression. Yan et al. confirmed that ubiquitin and proteasome-mediated ERAD is a primary check on K_ATP_ channels during biogenesis and that this process in part regulates the surface expression of K_ATP_ channels as inhibition of proteasome function led to an increase in surface expression of the channel (Yan et al., 2005). More recently, Wang and colleagues showed that Derlin-1, an ER membrane protein involved in recognition or retrotranslocation of substrates out of the ER for ERAD (Lilley and Ploegh, 2004; Ye et al., 2004), forms a complex with SUR1 and Kir6.2 and is also an important factor determining surface levels of K_ATP_ channels (Wang et al., 2012). SUR1 is a glycoprotein, containing two N-linked glycosylation sites. Conti et al. mutated these sites in SUR1 and observed ER retention of the protein, suggesting that lectin chaperones calnexin/calreticulin, which are known to assist the folding of glycoproteins, participate in the folding and assembly of K_ATP_ channels (Conti et al., 2002). Yan et al. also demonstrated that the molecular chaperone Hsp90 participates in the folding of K_ATP_ channels by interacting with SUR1, and that knockdown of Hsp90 reduced surface expression of the channel (Yan et al., 2010). These studies only begin to address the sequence of events and the mechanisms that govern the folding/assembly and degradation of the K_ATP_ channel, and represent important first steps in understanding this critical aspect of K_ATP_ channel biology.

### Assembly domains

A central question in understanding channel biogenesis is what are the assembly domains on the subunits themselves that direct this process? Using chimeras of Kir2.1, which is known to not associate with SUR1, and Kir6.2, Giblin et al. looked for minimal domains necessary for interactions between Kir6.2 and SUR1 (Giblin et al., 1999). They found that a proximal region (amino acids 208–279) in the Kir6.2 C-terminus is necessary for co-immunoprecipitation of the two subunits but this region is not sufficient to mediate formation of K_ATP_ functional channel complexes as no channel activity could be detected at the cell surface. Schwappach et al., with a similar approach using chimeras of Kir6.2 and Kir2.1 demonstrated the M1 and N-terminus of Kir6.2 are involved in K_ATP_ channel assembly and gating (Schwappach et al., 2000).

One particularly interesting assembly domain of the K_ATP_ channel is TMD0, the first bundle of transmembrane helices in SUR1. This domain, coupled with the first intracellular loop L0, is largely unique to SUR proteins; most eukaryotic ABC transporters have only the core structure of TMD1 and TMD2 with the two intracellular nucleotide binding domains NBD1 and NBD2 (Tusnady et al., 1997). Using chimeric SUR1 proteins containing TMD0 from another ABC transporter MRP1 known to not interact with Kir6.2, Schwappach et al. first demonstrated a role of TMD0 in mediating subunit interactions between Kir6.2 and SUR1 and in promoting forward trafficking of Kir6.2 (Schwappach et al., 2000). Subsequent work by others showed that in truncated Kir6.2 subunits (see below), TMD0 alone will increase surface expression of Kir6.2 and produce so-called “mini-K_ATP_” channels that display single-channel kinetics similar to WT channels, but are unresponsive to metabolic signals and pharmacological ligands (Babenko and Bryan, 2003; Chan et al., 2003). Studies utilizing naturally occurring TMD0 mutations present in patients with CHI have identified residues which may be crucial for folding or subunit interactions (Chan et al., 2003; Yan et al., 2004, 2007; Pratt et al., 2011), but the precise nature of the interface of TMD0 with Kir6.2 remains to be elucidated.

### Exiting the ER

As mentioned, forward trafficking and surface expression of K_ATP_ channels relies on assembly of both subunits in the ER. This makes Kir6.2 unique among Kir channels, thereby prompting studies to define the domain responsible for ER retention of unassembled Kir6.2 subunits. Tucker et al. first demonstrated that C-terminal truncations of 25 or 36 amino acids in Kir6.2 allowed for potassium currents in the absence of SUR1 (Tucker et al., 1997). Later work by Zerangue et al. utilized this seminal discovery in mapping the particular retention motif within the C-terminus of Kir6.2 and identified a tripeptide RKR localization signal (Zerangue et al., 1999) which was found to also function in SUR1 (Figure 2). The current model suggests these motifs must be masked during the assembly of the channel complex to allow forward trafficking to proceed. Unassembled or misassembled subunits are thus prevented from reaching the plasma membrane, allowing for quality control in the K_ATP_ biogenesis pathway. Questions still remain regarding the mechanism of how the RKR motifs function in ER retention. A study by Yuan et al using an artificial reporter construct first brought forth a model whereby an interplay between the 14-3-3 family of proteins and the coatamer complex 1 (COPI) acts to regulate ER to Golgi trafficking via interactions with the RKR motifs (Yuan et al., 2003). Their group showed that COPI proteins can specifically bind the RKR motif in Kir6.2. The same study demonstrated 14-3-3 proteins can also recognize the signal when multiple subunits are present. The model proposes that COPI proteins recognize RKR motifs on misassembled or unassembled subunits and promote their retrieval to the ER. The 14-3-3 proteins, by contrast, act as sensors for assembled subunits, and can bind properly assembled complexes and prevent recognition of their RKR motifs by COPI, allowing forward trafficking to proceed. A later study from the same lab using the 14-3-3 scavenger approach further substantiated a role of 14-3-3 proteins in regulating trafficking and surface expression of heterologously and endogenously expressed K_ATP_ channels (Heusser et al., 2006).

Anterograde, or forward trafficking signals on K_ATP_ channel subunits have been more challenging to define. While TMD0 clearly facilitates Kir6.2 expression, it can only do so when co-expressed with Kir6.2ΔC26, a C-terminal deletion construct missing the last 26 amino acids, in which the RKR motif resides (Chan et al., 2003). TMD0, therefore, is not the domain shielding the RKR signals from COPI proteins. Sharma et al. (1999) identified a putative forward trafficking signal in the distal C-terminus of SUR1 by making SUR1 constructs missing varying numbers of residues in the C-terminus (Sharma et al., 1999). However, another study showed that larger deletions in the SUR1 C-terminus had no effect on channel expression, making the existence of the C-terminal forward trafficking signal debatable (Giblin et al., 2002). No studies to date have resolved this issue or confirmed the existence of a *bona fide* anterograde signal on either subunit.

Interestingly, studies examining the kinetics of the biogenesis pathway using metabolic pulse-chase labeling have demonstrated the intrinsic inefficiency of K_ATP_ assembly, estimating that only about 20% of newly synthesized SUR1 or Kir6.2 actually forms mature complexes, with the remaining pool being rapidly degraded (Yan et al., 2004, 2005, 2010; Chen et al., 2011). CFTR and other ABC transporters also display a similar level of efficiency (Ward and Kopito, 1994). Yan et al. have shown that both SUR1 and Kir6.2 exhibit a biphasic degradation profile when expressed alone, each containing a fast and a slow component (Yan et al., 2004, 2005). Crane and Aguilar-Bryan also observed biphasic degradation of Kir6.2 expressed alone; however, they reported remarkable stability of SUR1 expressed alone (~25 h) (Crane and Aguilar-Bryan, 2004). These differences may be due to technical reasons or experimental conditions. Nevertheless, both groups saw increased stability of SUR1 and Kir6.2 when the two subunits were co-expressed, suggesting the subunits become more stable upon mulitimeric assembly (Crane and Aguilar-Bryan, 2004; Yan et al., 2005). While some differences are yet to be resolved, studies like these address fundamental aspects of K_ATP_ channel biology not by looking at snapshots, but by getting the dynamics involved in the biogenesis pathway. Many questions remain unanswered: How are nascent polypeptides recognized in the cytosol and translocated in the ER? What is the sequence of events involving folding and assembly of K_ATP_ channel subunits? Is folding co- or post-translational? What protein-protein interactions occur in the ER that guide this process, and what molecular chaperones are involved? What routes do K_ATP_ channels take after exiting the ER? Obviously much work needs to be done, but answers to these questions will provide a deep level of insight into how large, multimeric membrane proteins are assembled, and importantly, will allow researchers to more fully understand mechanisms of proteostasis and trafficking diseases, like CHI or CF.

## Gating regulation of K_ATP_ channels

It is well-understood that the primary mode of physiological regulation of K_ATP_ channel function is through the opposing inhibitory action of ATP and stimulatory action of MgADP. The bulk ATP concentration is relatively stable in most cells, however, raising doubt that ATP can serve as a primary regulator of the channel. In β-cells, ATP concentrations have been estimated to change from 2 to 4 mM when glucose is elevated from 0 to 10 mM (Detimary et al., 1998); such high levels of ATP are expected to prevent channel opening even at the lowest glucose concentrations. However, the small changes in [ATP], coupled with much larger changes in ADP levels in the opposite direction as glucose concentrations fluctuate, result in significant changes in the [ATP]/[ADP] ratio (Nilsson et al., 1996; Detimary et al., 1998) to shift the apparent ATP sensitivity and effectively regulate channel activity (Tarasov et al., 2004). Thus, when glucose levels are low, MgADP stimulation will predominate, K_ATP_ channels will be open, the cell will be hyperpolarized, and no insulin will be secreted. When glucose levels are high, ATP inhibition will take over, closing K_ATP_ channels, causing membrane depolarization, opening voltage-gated Ca^2+^ channels and triggering insulin exocytosis.

In the absence of ATP, K_ATP_ channels display so-called burst kinetics, characterized by periods of rapid openings and closings separated by long closed intervals (Alekseev et al., 1997). ATP inhibition of K_ATP_ channels, involving non-hydrolytic binding at the intracellular face of Kir6.2, acts to decrease the frequency and length of the burst periods, and increases the duration of the closed intervals (Babenko et al., 1999). Based on mutagenesis and docking studies using homology models of Kir6.2, binding is thought to occur at an interface involving the N- and C-domains of one subunit, and the N-domain of an adjacent Kir6.2 subunit (Antcliff et al., 2005). Each channel contains four ATP-binding sites, yet ATP binding at a single site is sufficient to close the channel, in support of a concerted gating model (Markworth et al., 2000; Drain et al., 2004). Interestingly, however, SUR1 sensitizes Kir6.2 to ATP inhibition by about a factor of 10 (Tucker et al., 1997). This could be due to allosteric effects on Kir6.2 or facilitation of ATP binding by SUR1 (Babenko, 2005); at this point the mechanism remains unclear.

Nucleotide activation occurs at the nucleotide binding domains of SUR1 and acts to antagonize ATP inhibition (Gribble et al., 1997). The requirement of Mg^2+^ in this process has prompted studies to examine nucleotide interactions and hydrolysis at the NBDs. These studies led to a proposal that hydrolysis of MgATP to MgADP at NBD2, which contains the consensus ATPase site, stabilizes ATP binding at NBD1, which carries a degenerate ATPase site, and drives dimerization of the NBDs to promote channel opening at Kir6.2 (Zingman et al., 2001; Matsuo et al., 2005; Masia and Nichols, 2008). It is worth noting that direct measurements of MgATP hydrolysis using purified SUR or recombinant SUR NBDs indicate relatively poor hydrolysis efficiency (Masia et al., 2005; De Wet et al., 2007), suggesting that increased MgADP binding at NBD2 as intracellular [ADP] rises may be sufficient to induce or stabilize conformational changes at the NBDs to stimulate channel opening. Recent work by Ortiz et al. using glibenclamide binding to probe switching of the NBDs between closed and open dimer conformations supports the notion that nucleotide hydrolysis at NBD2 is not required for conformational switch (Ortiz et al., 2012, 2013). Physiologically, the regulation of SUR1 in response to an increase in intracellular [ADP] is critical for repolarizing the β-cell when glucose levels drop; without it the β-cell will be unable to stop secreting insulin. This is one common mechanism for mutations identified in SUR1 in patients with CHI (Nichols et al., 1996a; Shyng et al., 1998), who persistently secrete insulin even under severely low blood glucose levels.

Membrane phosphoinositides, such as phosphatidylinositol-4,5-bisphosphate (PIP_2_), and metabolic derivatives of free fatty acids, long-chain acyl-CoA esters (LC-CoAs) also have profound effects on K_ATP_ channel gating (Baukrowitz et al., 1998; Branstrom et al., 1998; Shyng and Nichols, 1998). Both PIP_2_ and LC-CoAs interact with Kir6.2 via similar mechanisms to increase channel open probability and antagonize ATP inhibition (Schulze et al., 2003). Mutagenesis studies indicate that PIP_2_ and ATP may have overlapping but non-identical binding sites in Kir6.2 (Shyng et al., 2000; Cukras et al., 2002a,b; Antcliff et al., 2005). While Kir6.2 interacts directly with PIP_2_, SUR1 increases the sensitivity of the channel to PIP_2_ stimulation (Baukrowitz et al., 1998; Shyng and Nichols, 1998). Recent work by Pratt et al. shows that TMD0 of SUR1, which is known to increase the open probability of Kir6.2, does so by stabilizing the interaction between Kir6.2 and PIP_2_ (Pratt et al., 2011). A crucial role of PIP_2_ in maintaining channel activity in β-cells has been clearly demonstrated (Lin et al., 2005); mutations which disrupt the channel response to PIP_2_ have been identified in patients with CHI (Lin et al., 2006b, 2008). The relevance of PIP_2_ or LC-CoAs to physiological regulation of K_ATP_ activity however, is less certain (Tarasov et al., 2004), although pathological changes in LC-CoAs have been proposed to impact channel function (Larsson et al., 1996; Riedel and Light, 2005).

## Pharmacological regulation of K_ATP_ channels

An important class of agents which regulate K_ATP_ channels are sulfonylureas (Aguilar-Bryan and Bryan, 1999; Gribble and Reimann, 2003b). Discovered in the 1940s as sulfonamide drugs that have hypoglycemic effects, sulfonylureas interact specifically with the sulfonylurea receptor SUR, giving it its namesake. Sulfonylureas have been used in the treatment of non-insulin dependent diabetes for decades, yet precisely how they promote insulin release in the body remained a mystery for years. It is now well understood that sulfonylureas bind directly to SUR1 and inhibit channel activity, thus stimulating insulin release by depolarizing the β-cell independent of glucose. Tolbutamide was part of the first generation of hypoglycemic compounds, later replaced by second-generation compounds like glibenclamide, which have 100 to 1000-fold more potency in blocking K_ATP_ currents. A related group of sulfonamide compounds, most notably diazoxide, also bind specifically to SUR1 but activate channels (Dunne et al., 1989; Moreau et al., 2000). Diazoxide is part of a diverse group of drugs that stimulate K^+^ currents, known as potassium channel openers (KCOs) (Manley et al., 1993).

Although the K_ATP_ channel was first identified in cardiac myocytes by Aki Noma in 1983 (Noma, 1983), its molecular identify remained uncertain for many years. Sulfonyulureas, owing to their specificity, were used as probes in the purification and cloning of K_ATP_ channels. Early patch-clamp experiments demonstrated that sulfonylureas were specific blockers of β-cell K_ATP_ channels, while diazoxide specifically opened them. A significant advancement came from using labeled derivatives of glibenclamide, [^3^H]glibenclamide (Kramer et al., 1988), and later [^125^I]iodoglibenclamide (Aguilar-Bryan et al., 1990), which could label a 140-kDa protein (SUR1) in isolated membranes of various β-cell lines and led to cloning of the SUR1 gene (Aguilar-Bryan et al., 1995). Schwanstecher et al. further used ^125^I-iodo-azidoglibenclamide, an azido analog of glibenclamide, and found it would co-photolabel a 38 kDa protein in addition to SUR1 and with the same apparent K_D_ (Schwanstecher et al., 1994); this species was later identified as Kir6.2 (Inagaki et al., 1995). This provided the first evidence that the K_ATP_ channel is a multimeric complex of Kir6.2 and SUR1.

Significant progress has since been made in defining the precise mechanism by which sulfonylureas and glinides, another class of K_ATP_ channel inhibitors used in diabetes therapy, block K_ATP_ channel function. All sulfonylureas and glinides demonstrate high and low-affinity components of inhibition, such that their dose-response curves are generally biphasic, with variable separation between the two sites (Gribble and Reimann, 2003a). At low concentrations, interaction with the SUR subunit results in 50–75% reduction in current amplitude when administered to the cytoplasmic face of membrane patches, suggesting that channels can remain open to an extent when bound to the drug. The low-affinity component is attributed to interaction with Kir6.2, but this only occurs at very high drug concentrations.

**Figure 3:**
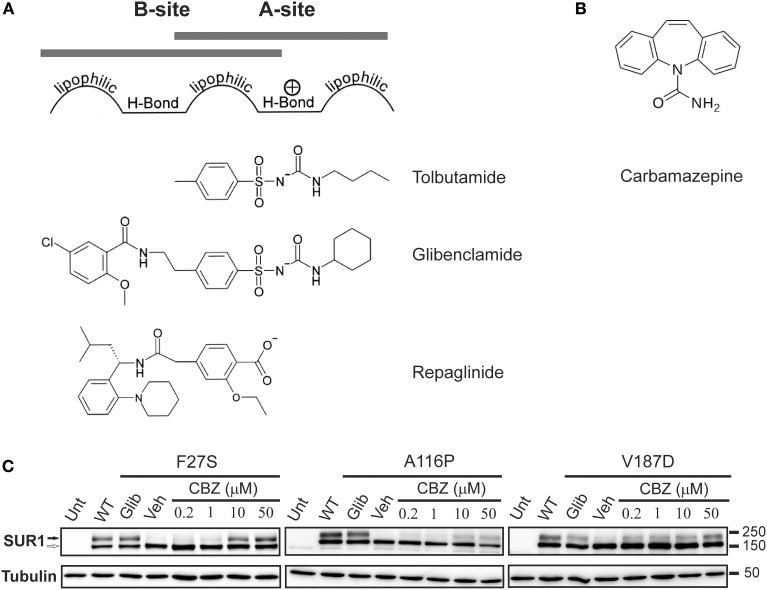
**The processing defect of SUR1 caused by TMD0 trafficking mutations is corrected by glibenclamide and carbamazepine. (A)** Pharmacophore model for binding of various K_ATP_ channel blockers that function as effective pharmacological chaperones, showing the chemical moieties thought to confer affinity for either site A or site B on SUR1. **(B)** Chemical structure of carbamazepine. **(C)** Western blot of whole cell lysates of COSm6 cells transiently transfected with Kir6.2 plus WT or TMD0 mutant SUR1 cDNAs. SUR1, a glycoprotein, shows two bands in immunoblots: a lower core glycosylated (immature; open arrow) form that has not exited the ER and an upper complex-glycosylated (mature; solid arrow) band that has trafficked through the Golgi. Incubation of cells with glibenclamide (5 μM; Glib) or carbamazepine (CBZ) for 16 h increased levels of the mature band of TMD0 trafficking mutants compared to those treated with DMSO (veh). The effect of carbamazepine was dose-dependent, with an effect detectable at a concentration as low as 0.2 μM. Untransfected control is shown for comparison. Adapted from Figure 1 in Chen et al. (2013b).

According to the pharmacophore model, structurally diverse compounds can possess overlapping electronic and stereochemical properties that allow them to bind a common receptor site. SUR was proposed to bind sulfonylureas accordingly with a bipartite binding pocket (Figure 3A). Consistent with this, the enhanced affinity observed in glibenclamide is attributed to interaction with the two overlapping binding sites on SUR1, termed site A and site B (Brown and Foubister, 1984). Site A is proposed to interact with a lipophilic group adjacent to the negative charge of a sulfonylurea group, while site B recognizes a lipophilic group adjacent to an amide. Glibenclamide possesses both of these moieties, while most other sulfonylureas and hypoglycemic agents such as glinides possess one or the other. Sulfonylureas are further distinguished by their ability to block either SUR1 or SUR2 K_ATP_ channels. Compounds that contain a non-sulfonylurea moiety (such as glibenclamide, glimepiride, repaglinide) can bind with high affinity to SUR1 and SUR2, while those with only the core sulfonylurea structure (tolbutamide, gliclizide) are specific for SUR1 (Quast et al., 2004). This difference made tolbutamide a pharmacological tool to uncover the sulfonylurea binding (A) site. Research using chimeric receptors of SUR1/SUR2 showed that TMD2 of SUR1 is critical for sulfonylurea binding. Further studies showed that mutation of S1237 of the cytoplasmic loop between TMs 15 and 16 to tyrosine (the equivalent residue in SUR2) abolished high affinity tolbutamide block and [^3^H]glibenclamide binding (Ashfield et al., 1999) [note that S1237 in SUR1 is now referred to as S1238, as current sequence information for *ABCC8* (GenBank reference NM_00352.2) includes the alternate exon 17 (GenBank L78208) which contains an additional amino acid]. Further, the reciprocal mutation in SUR2 (Y1206S) increased [^3^H]glibenclamide binding by roughly 10-fold. Mapping of site B in SUR1 came from a study examining affinity labeling with [^125^I]iodoglibenclamde which identified a ~50 kDa fragment including TMD0-L0 (Bryan et al., 2004). A more precise definition came from Vila-Carriles et al. by using deletions and alanine scanning in TMD0 and L0 of SUR1 to monitor photolabeling by [^125^I]azido-iodoglibenclamide (Vila-Carriles et al., 2007). The authors concluded that L0 of SUR1 is absolutely essential for glibenclamide binding, while TMD0 is nonessential. Scanning alanine mutations showed that Y230 and W232 are critical for high affinity photolabeling. Further, they determined that the N-terminal 33 amino acids of Kir6.2 are involved in site B labeling by [^125^I]azido-iodoglibenclamide. Together, these results indicate that site A (TMD2) and site B (L0) are within close physical proximity such that they can cooperatively bind glibenclamide, and that the N-terminus of Kir6.2 also forms part of the site B binding pocket.

## Pharmacological regulation of K_ATP_ channel trafficking defects

Over the past 10 years, it has been recognized that, in addition to their action as specific K_ATP_ channel blockers, sulfonylureas also promote the proper folding and biogenesis of trafficking-impaired mutant SUR1 proteins identified in patients with CHI by acting as pharmacological chaperones. They achieve this with domain specificity, only rescuing mutations within TMD0 of SUR1. In principle, this opened up a new therapeutic avenue for sulfonylureas, in which they could be administered to patients with TMD0 trafficking mutations and rescue K_ATP_ channel function in β-cells. Sulfonylureas are imperfect correctors, however, as they often irreversibly block channel function of rescued mutant channels. This is an unsuitable therapeutic approach for treating diseases like CHI, which require functional channels at the cell surface in order to limit insulin release and restore blood glucose to normal levels. Recently, additional new compounds such as carbamazepine (CBZ) (Figure 3B), which also correct trafficking-impaired K_ATP_ channels with mutations in TMD0 of SUR1 without the irreversible block observed with glibenclamide, have been identified. As discussed below, *in vitro* studies with pharmacological chaperones have increased our understanding of how certain K_ATP_ channel mutations lead to disease, strengthening the link between genotype and phenotype, while also highlighting general principles involved in the gating and molecular assembly of heteromeric ion channels, like K_ATP_.

## K_ATP_ channel trafficking defects in human disease

Proper cellular function relies on both the absolute number and subcellular localization of many proteins; this is particularly true of membrane proteins like K_ATP_. For ion channels and other membrane or secreted proteins, translation begins in the cytosol but soon becomes associated with the endoplasmic reticulum (ER). Here it is thought the bulk of protein folding and quaternary assembly take place. This occurs under the auspices of an array of molecular chaperones, integral members of the ER quality control system, which ensure the proper folding, processing, and structural integrity of nascent proteins while preventing the accumulation of defective proteins which may disrupt normal cell function. Trafficking mutations can interfere with this process by disrupting protein folding and molecular assembly in the ER, generally leading to retention and clearance of mutant proteins through ERAD.

Mislocalization of cell-surface proteins which are otherwise functional has been demonstrated in numerous diseases, including CF, familial hypercholesteremia, retinitis pigmentosa, and diabetes insipidus (Powers et al., 2009). In CF, the ΔF508 deletion (present in 90% of CF patients) in CFTR leads to ER retention and rapid degradation of the incompletely processed protein by the proteasome (Jensen et al., 1995; Ward et al., 1995). Insufficient levels of this chloride channel at the cell surface prevent cAMP-mediated chloride ion, water, and bicarbonate conductance in a variety of tissues. More recently, it has been established that point mutations in the genes encoding the K_ATP_ channel subunits can also interfere with or prevent proper folding and/or molecular assembly in the ER (Table 1). These mutations are found throughout the SUR1 and Kir6.2 proteins. Normal levels of protein are usually translated, but mutant proteins are unable to exit the ER, and as with ΔF508-CFTR and many other conformationally-defective proteins, are degraded through the ubiquitin/proteasome pathway. K_ATP_ channel trafficking mutations have since been shown to be a common mechanism in CHI (Cartier et al., 2001; Taschenberger et al., 2002; Crane and Aguilar-Bryan, 2004; Tornovsky et al., 2004; Yan et al., 2004, 2007; Marthinet et al., 2005; Muzyamba et al., 2007; Fukuda et al., 2011; Powell et al., 2011; Faletra et al., 2013), in which loss of K_ATP_ channels at the cell surface results in unregulated insulin secretion following constitutive depolarization of the β-cell.

**Table 1:** **Congenital Hyperinsulinism-associated K_ATP_ channel trafficking mutations and response to pharmacological rescue^1^**.

**Mutation**	**Domain**	**Rescue by SU**	**Rescue by CBZ**	**Gating property**	**References**
**SUR1**					
G7R	TMD0	Yes	Yes	Normal	Yan et al., 2007
N24K	TMD0	Yes	Yes	Normal	Yan et al., 2007
F27S	TMD0	Yes	Yes	Normal	Yan et al., 2007
R74W	TMD0	Yes	Yes	ATP-insensitive	Yan et al., 2007
A116P	TMD0	Yes	Yes	Normal	Yan et al., 2004
E128K	TMD0	Yes	Yes	ATP-insensitive	Yan et al., 2007
V187D	TMD0	Yes	Yes	Normal	Yan et al., 2004
R495Q	TMD1	Yes	Yes	Unknown	Yan et al., 2007
E501K	TMD1	Yes	Yes	Unknown	Yan et al., 2007
L503P	TMD1	No	No	Unknown	Yan et al., 2007
F686S	NBD1	No	No	Unknown	Yan et al., 2007
G716V	NBD1	No	No	Unknown	Yan et al., 2007
E1324K	TMD2	N.D.^3^	N.D.	Normal	Faletra et al., 2013
L1350Q	NBD2	No	No	Unknown	Yan et al., 2007
ΔF1388^2^	NBD2	No	No	MgADP-insensitive	Cartier et al., 2001
M1395R	NBD2	N.D.	N.D.	Unknown	Faletra et al., 2013
R1419H	NBD2	No	No	Unknown	Tornovsky et al., 2004
R1437Q	NBD2	No	No	Unknown	Muzyamba et al., 2007
D1472H	NBD2	No	No	Unknown	Yan et al., 2007
R1494W	NBD2	No	No	Unknown	Tornovsky et al., 2004
L1544P^2^	NBD2	No	No	MgADP-insensitive	Taschenberger et al., 2002
**Kir6.2**					
W91R	M1	N.D.	N.D.	Unknown	Crane and Aguilar-Bryan, 2004
H259R	C-term	N.D.	N.D.	Unknown	Marthinet et al., 2005
E282K	C-term	N.D.	N.D.	Unknown	Taneja et al., 2009
R301G	C-term	N.D	N.D	Inactivation^4^	Lin et al., 2008
R301H	C-term	N.D	N.D	Inactivation	Lin et al., 2008
R301P	C-term	N.D	N.D	Inactivation	Lin et al., 2008

1 *Only published mutations that have been tested for surface expression are included*.

2 *These mutations were rescued to the cell surface by mutating the RKR ER retention signal to AAA*.

3 *N.D.: Not determined*.

4 *Inactivation: Spontaneous current decay in the absence of inhibitory ATP*.

Among the mutations documented, A116P- and V187D-SUR1, both located in TMD0, exhibited reduced association with Kir6.2 in co-immunoprecipitation experiments (Chan et al., 2003), supporting a role of TMD0 in subunit-subunit interactions. Further, ΔF1388-, A116P-SUR1 and W91R-Kir6.2 all showed accelerated degradation (Crane and Aguilar-Bryan, 2004; Yan et al., 2004, 2005). These observations are consistent with the mutant proteins being misfolded and/or unable to form channel complexes. Of note, the aforementioned studies were all conducted in mammalian cells rather than *Xenopus oocytes* as oocytes cultured at a lower temperature have less stringent ER quality control and allow surface expression of mutant channels that would otherwise be retained intracellularly (Drumm et al., 1991). In addition to protein misfolding, recently a Kir6.2 mutation E282K identified in a case of histological focal CHI (due to combination of a paternal K_ATP_ mutation and clonal loss of heterozygosity of 11p15) was reported to diminish surface expression of K_ATP_ channels by disrupting a di-acidic ER exit signal in Kir6.2 involved in concentration of the channel protein into COPII vesicles without affecting channel protein folding (Taneja et al., 2009). Also, the SUR1 mutation R1394H reportedly causes retention of the channel in the Golgi compartment of HEK239 cells, thereby preventing surface expression (Partridge et al., 2001). Although increased internalization and degradation of surface channels may also reduce the number of channels at the cell surface no such examples have been reported.

## Correction of K_ATP_ channel trafficking defects by pharmacological chaperones

The discovery that protein misfolding is an important cause of various diseases has prompted intense investigation into ways of overcoming the biogenesis defect as a means of therapy (Powers et al., 2009). A key finding came from Denning et al, who showed that cells expressing ΔF508-CFTR had greater surface levels of the channel when grown at reduced temperature (Denning et al., 1992). This demonstrated that manipulation of the cellular folding environment can positively impact the biogenesis of proteins. Certain exogenous compounds, like glycerol or DMSO, were also shown to enhance the expression of trafficking-impaired proteins, acting as chemical chaperones (Brown et al., 1996). It is thought these compounds interact directly with the protein during folding and assembly in the ER and impact biogenesis by one or several of the following mechanisms: (1) reducing degradation by thermodynamically stabilizing a misfolded conformation of the protein; (2) increasing the folding rate by stabilizing a folding intermediate; (3) decreasing the misfolding rate by stabilizing the native state. Alternatively, chemical chaperones could indirectly affect biogenesis of proteins by interacting with endogenous molecular chaperones in the ER.

Chemical chaperones, such as glycerol, are nonspecific, however, and enhance the expression of multiple proteins in a cell. In a seminal study, Loo et al. demonstrated that pharmacological ligands can specifically promote the stability and expression of trafficking-impaired mutant isoforms of P-glycoprotein (P-gp), a multidrug resistant protein (Loo and Clarke, 1997). Multiple drug compounds known to directly interact with P-gp rapidly enhanced expression of various engineered P-gp trafficking mutants in a dose-dependent manner, producing functional proteins at the plasma membrane. These results were soon echoed by various other groups using high-affinity ligands for lysosomal α-galactosidase A (Fan et al., 1999), the V_2_ vasopressin receptor (Morello et al., 2000), the HERG (human *ether-a-go-go*-related gene) potassium channel (Zhou et al., 1999), among others. An emerging theme began to develop in which specific chemical chaperones, termed pharmacological chaperones, could be administered therapeutically in order to reverse trafficking defects found in patients with complex diseases. High-throughput screens have identified more advanced compounds which can rescue mutants with more subtle trafficking defects. Several promising hits are currently undergoing clinical trials for treatment of lysosomal storage diseases, including Fabry's, Pombe's, Tay-Sachs, and Gaucher's diseases, as well as CF. Discussed below are recent studies describing the functional restoration of trafficking-impaired K_ATP_ channels with the use of small molecule correctors. These studies have added to our fundamental understanding of the biogenesis of K_ATP_ channels while bringing forth a new therapeutic route for treating CHI. Further, they have led to new understanding in the ways that pharmacological ligands interact with K_ATP_ channels and impact their function.

### Correction of K_ATP_ channel trafficking defects by sulfonylureas

As with many diseases, a major challenge has been to define how mutations in K_ATP_ channel subunits associated with CHI promote disease. Some mutant channels had been shown to be unresponsive to MgADP stimulation (Nichols et al., 1996b; Shyng et al., 1998; Snider et al., 2013), while others produce non-functional truncated proteins due to insertion of a stop codon (Nestorowicz et al., 1997, 1998). An important discovery came from Cartier et al., who showed that one K_ATP_ channel mutation identified in CHI, ΔF1388 in SUR1, prevented surface expression of the channel when expressed in COS cells (Cartier et al., 2001). Interestingly, functional channel complexes were observed when the ER retention signal RKR in ΔF1388-SUR1 was mutated to AAA, which can often allow some trafficking-impaired proteins to escape the ER. This placed some forms of CHI now in the same category as CF and other protein misfolding diseases, whereby loss-of-function results from mislocalization of mutant proteins. Subsequent work identified additional SUR1 mutations in CHI patients that impair the proper trafficking of K_ATP_ channels, including L1544P, A116P, and V187D (Taschenberger et al., 2002; Yan et al., 2004).

The successful use of pharmacological ligands in correcting trafficking defects of other membrane proteins prompted several groups to apply that same principle to trafficking-impaired K_ATP_ channels. Yan et al. (2004) demonstrated that two CHI mutations, A116P and V187D, both located in the first transmembrane domain TMD0 of SUR1, could be rescued by sulfonylureas *in vitro*. Functional study of mutant channels rescued to the cell surface by the reversible sulfonylurea tolbutamide revealed normal sensitivity to MgADP and ATP once tolbutamide was washed out, suggesting that trafficking mutations may not interfere with channel function. Presumably, as is the case for pharmacological rescue of other trafficking-impaired proteins, sulfonylureas facilitate the biogenesis of these SUR1 mutants by interacting with the protein directly during folding and assembly in the ER. Yet an understanding of the mechanism of the trafficking impairment is key to grasping the mechanism of recovery. Previously, Chan et al. showed that TMD0 domain of SUR1 harboring the A116P or V187D mutation, had reduced association with Kir6.2 in co-immunoprecipitation experiments (Chan et al., 2003). As TMD0 is known to mediate interactions between SUR1 and Kir6.2, it is reasonable to assume that mutations in TMD0 disrupt these subunit interactions and prevent channel trafficking out of the ER. Yan et al. showed, however, that the trafficking defect in A116P and V187D is intrinsic to SUR1. This is based on the observation that in the absence of Kir6.2, A116P and V187D also prevented Kir6.2-independent surface expression of a SUR1 protein in which the RKR ER retention signal is inactivated by mutation to AAA (SUR1_AAA_). Another potential explanation for the trafficking defect is that these mutations may prevent proper shielding of the RKR ER retention signals that must occur during assembly in the ER, as has been demonstrated for the L1544P mutation (Taschenberger et al., 2002). Yet mutation of these signals in both subunits also failed to improve surface expression of the A116P or V187D mutants. These results suggest that SUR1 misfolding, which also likely adversely affects association with Kir6.2, prevents exit of these mutants from the ER. Consistent with this notion, channel trafficking defects caused by A116P and V187D could be overcome by culturing cells at lower temperature (Yang et al., 2005), a condition known to facilitate protein folding. Metabolic pulse-chase experiments demonstrated that glibenclamide slowed A116P-SUR1 degradation even in the absence of Kir6.2 and promoted maturation of the mutant SUR1 when Kir6.2 was co-expressed (Yan et al., 2004), providing evidence that sulfonylureas facilitate folding and/or prevent misfolding of mutant channels during assembly in the ER.

The question remained, however, of whether sulfonylureas act as true pharmacological chaperones by promoting biogenesis through direct binding of the channel. Compelling evidence came from a study showing that mutation of residues critical for sulfonylurea and glinide binding abolished or reduced these compounds' ability to rescue K_ATP_ channel trafficking mutants (Yan et al., 2006). Binding of tolbutamide, a first generation sulfonylurea, has been shown to depend on S1238 in SUR1, representing site A (Ashfield et al., 1999). Accordingly, mutation of S1238 to tyrosine abolished tolbutamide rescue of SUR1 mutants A116P and V187D. Glibenclamide binding involves both site A (S1238) and site B, which includes residue Y230 (Bryan et al., 2004). Mutation of either site A (S1238Y) or site B (Y230A) diminished glibenclamide's rescue effect, while simultaneous mutation of both completely abolished it, suggesting that the sulfonylurea and benzamido moieties both contribute to the rescue effect of glibenclamide. Interestingly, the site B mutation Y230A, in addition to attenuating the effect of glibenclamide and abolishing the effect of rapaglinide, also rendered tolbutamide ineffective at rescuing mutant channels. This suggests that either Y230 is involved in tolbutamide binding or Y230 is necessary for post-binding events involved in tolbutamide rescue, such as coupling of SUR1 and Kir6.2 subunits. In support of the latter, Y230 has been shown to be in close proximity to the N-terminus of Kir6.2 (Vila-Carriles et al., 2007). Further, deletion of the Kir6.2 N-terminus abolishes tolbutamide channel block in membrane patches (Koster et al., 1999; Reimann et al., 1999), suggesting a functional interface at Y230 of SUR1 and the N-terminus of Kir6.2 that couples tolbutamide binding to changes in channel activity. The results of S1238Y and/or Y230A mutants on the ability of sulfonylureas to rescue K_ATP_ trafficking mutants were also in parallel to their ability to block channel activity. Thus, there is likely a common mechanism in transducing ligand binding to functional outcome. Finally, a key finding from this study is that sulfonylureas act on the channel complex, rather than SUR1 alone, to restore surface expression of trafficking mutants, as pharmacological rescue by sulfonylureas is dependent on Kir6.2. Expression of either A116P_AAA_-SUR1 or V187D_AAA_-SUR1, which lack the RKR ER retention motif, could not be rescued without co-expression of Kir6.2. The requirement of Kir6.2 for the rescue effect could be explained by the involvement of Kir6.2 in sulfonylurea binding to SUR1; alternatively, Kir6.2 could participate in the tertiary folding of mutant SUR1 subunits.

An interesting trend noted for all K_ATP_ trafficking mutants tested thus far is that only mutations within TMD0 of SUR1 are amenable to pharmacological rescue by sulfonylureas (Table 1). TMD0, the first of three transmembrane domains in SUR1, is a unique structural feature not shared by most ABC transporters, including CFTR or P-gp, which contain only TMD1 and TMD2. When expressed alone, TMD0 has been shown to physically associate with Kir6.2 and both facilitate its expression and modulate its gating function, making this domain a distinct functional entity (Babenko and Bryan, 2003; Chan et al., 2003). The studies described above demonstrate that sulfonylureas exert chaperoning effects by binding regions downstream of TMD0. Thus, ligand binding to the core ABC structure is likely translated into structural and functional outcomes at TMD0 to overcome trafficking defects caused by mutations within this region. Such substrate-induced transmembrane domain interactions have been reported previously: in human P-gp, also an ABC transporter, substrate binding promoted superfolding of partially folded intermediates via interactions between the two transmembrane domains TMD1 and TMD2 (Loo and Clarke, 1998). As such, a likely mechanism for the pharmacological chaperone effect is that sulfonylurea binding to the SUR1/Kir6.2 complex induces structural changes in TMD0 that restore functional interactions between SUR1 and Kir6.2, allowing trafficking of the channel out of the ER. The precise nature of the interface between TMD0 and Kir6.2 is unclear, however. Interestingly, a recent study by Zhou et al. showed that a point mutation in Kir6.2, Q52E, located in the N-terminus of the protein just before the slide helix, partially compensated for the trafficking defects caused by SUR1-TMD0 mutations F27S and A116P, indicating that altered molecular interactions with Kir6.2 can overcome impaired channel folding/assembly caused by TMD0 mutations (Zhou et al., 2013). An understanding of how these domains are interacting will provide valuable insight into not only the mechanism of pharmacological rescue, but also how physiological or pharmacological interactions at SUR1 are coupled to changes in channel activity at Kir6.2.

### Correction of K_ATP_ channel trafficking defects by ΔF508-CFTR correctors, in particular by carbamazepine

The fact that multiple TMD0 trafficking mutants have normal responses to metabolic signals and pharmacological ligands provides proof of principle that pharmacological rescue is a therapeutically viable alternative to the current treatment for many CHI with such mutations, which often relies on partial or near complete removal of the pancreas. Translation of these findings, however, has been hindered by the pharmacology of glibenclamide, the most effective sulfonylurea at rescuing trafficking-impaired K_ATP_ channels. SUR1 binds glibenclamide with high affinity and slow dissociation kinetics, resulting in an irreversible block on channel function; rescued channels would therefore be unable to hyperpolarize the β-cell in order to attenuate insulin release. Tolbutamide, another sulfonylurea that effectively rescues multiple TMD0 trafficking mutants, binds K_ATP_ channels reversibly. A study released by the University Group Diabetes Program, however, implicated tolbutamide in increased mortality secondary to cardiovascular events (Schwartz and Meinert, 2004). Thus, in terms of therapy, there is a need for additional compounds that promote robust recovery of K_ATP_ channel trafficking mutants and bind reversibly, but are also safe for administration to patients.

Like trafficking-impaired K_ATP_ channels in CHI, ΔF508-CFTR causes partial misfolding of the channel and clearance through the ubiquitin/proteasome pathway, resulting in CF. Much effort has been devoted to identifying small molecules that correct this trafficking defect, and high-throughput drug screens have yielded several promising compounds (Pedemonte et al., 2005; Carlile et al., 2007). As CFTR and SUR1 are both members of the ABC transporter superfamily and have common structural features in the ABC core domain, it is reasonable to hypothesize that small molecules which correct folding and trafficking defects in ΔF508-CFTR might also rescue trafficking-impaired K_ATP_ channels caused by mutations in SUR1. Powell et al. first reported the effects of compounds known to stimulate CFTR trafficking on human β-cells lacking functional K_ATP_ currents from CHI patients harboring various *ABCC8* mutations (Powell et al., 2011). Although it remains unknown how these mutations impact channel trafficking and gating to cause loss of channel activity, the study demonstrated that a ΔF508-CFTR corrector, 4-phenylbutyrate, could recover activities of K_ATP_ channels in β-cells isolated from a patient bearing the SUR1 compound heterozygous mutation Arg998X/Ser1449dup (Powell et al., 2011). Additionally, the study showed that incubation of β-cells from another patient bearing homozygous *ABCC8* intronic mutation c.1467+5G>A with a combination of 3-isobutyl-1-methylxanthine (IBMX), forskolin, and phorbol myristic acid (PMA), compounds expected to activate PKA and PKC, for 1 h also led to increased channel activity. Because PKA activation has been reported to promote trafficking of several ion channels to the cell surface whereas PKC activation has been reported to reduce surface K_ATP_ expression by diverting endocytosed channels to lysosomal degradation (Manna et al., 2010), the authors speculate that the positive effect of PKA activation likely overrides the negative effects of PKC to lead to an overall increase in surface expression of K_ATP_ channels (Powell et al., 2011). It is interesting to note that a role of PKA in K_ATP_ channel trafficking has indeed been reported. A study by Yang et al. showed that glucose stimulation recruits β-cell K_ATP_ channels to the cell surface in a PKA-dependent manner (Yang et al., 2007). In addition, Chen et al. showed that PKA activation promotes K_ATP_ channel trafficking to the surface in INS-1 rat insulinoma cells by promoting F-actin depolymerization without affecting overall channel protein levels (Chen et al., 2013a). The findings by Powell et al. will undoubtedly stimulate future research to harness the therapeutic potential of these compounds and to understand the molecular mechanisms by which these molecules restore functional expression of K_ATP_ channels in CHI patients.

A more recent study by Sampson et al. tested the effects of multiple CFTR correctors identified in a chemical library screen (Carlile et al., 2007) on the processing efficiency of two SUR1 trafficking mutants, A116P and V187D (Sampson et al., 2013). Among the compounds that improved the processing efficiency of the two mutant SUR1 proteins is the drug carbamazepine (CBZ). CBZ has been used for decades in the treatment of epilepsy, neuropathic pain, as well as mental illnesses like bipolar disorder. CBZ has a well-documented role as a sodium channel blocker, but studies have shown effects on calcium channels as well as GABA receptors (Ambrosio et al., 2002). Our group focused attention on CBZ as a potential pharmacological chaperone for SUR1 trafficking mutants, as it is approved by the Food and Drug Administration and has an established biosafety profile. In a recent report we demonstrated that CBZ effectively rescues multiple K_ATP_ trafficking mutations previously identified in CHI (Chen et al., 2013b) (Figure 3C). Interestingly, as with sulfonylureas, only mutations present in TMD0 of SUR1 were amenable to pharmacological rescue by CBZ. This suggests sulfonylureas and CBZ may act through a common mechanism to enhance surface expression of trafficking mutants, such as by stabilizing interactions between TMD0 and Kir6.2. There are studies showing, however, that CBZ can induce autophagy to facilitate clearance of misfolded protein aggregates (Sarkar et al., 2007; Hidvegi et al., 2010), which could in turn alleviate ER stress and promote surface expression of trafficking mutants. This raises the possibility that CBZ may enhance surface expression of TMD0 trafficking mutants not by acting as a pharmacological chaperone but by inducing autophagy. Disfavoring this idea, inhibition of autophagy by chloroquine or Ly294002 did not block CBZ's ability to rescue K_ATP_ trafficking mutants. Moreover, stimulation of autophagy by rapamycin or Li^+^ did not enhance mutant K_ATP_ channel processing or expression. These results indicate that CBZ most likely rescues mutant channels through an autophagy-independent pathway.

A surprising and intriguing finding by Chen et al. is that CBZ inhibits the activity of mutant channels rescued to the cell surface, as demonstrated in ^86^Rb^+^ efflux experiments. Subsequent electrophysiology experiments showed that CBZ inhibits channel activity when applied to the cytoplasmic face of isolated membrane patches containing wild-type K_ATP_ channels. These observations suggest that CBZ may act as a K_ATP_ channel antagonist, like sulfonylureas. Further characterization of the inhibitory effect of CBZ by ^86^Rb^+^ efflux assays showed that the function of rescued mutant channels could be completely recovered after extensive washout (~90 min), similar to that observed for the reversible, low-affinity sulfonylurea tolbutamide but not the irreversible high affinity sulfonylurea glibenclamide (Yan et al., 2004). Because the effect of CBZ on mutant channel trafficking could be detected at a concentration as low as 0.2μM (Chen et al., 2013b) (see Figure 3C), it is conceivable that low doses of CBZ could be used to rescue mutant channels to the cell surface without potent inhibition of channel activity. Another approach to circumvent the problem of chaperone inhibitors may be to simultaneously apply correctors and compounds that boost the function of rescued proteins, referred to as potentiators (Rowe and Verkman, 2013). Indeed, we have found that while diazoxide is unable to activate channels rescued by the irreversible antagonist glibenclamide, it significantly increased the activity of channels rescued by CBZ in Rb efflux assays. In these experiments, CBZ was not included in the 40-min efflux period so some CBZ was likely to have dissociated from the rescued channels, as evidenced by a small increase in efflux even without diazoxide; however, inclusion of diazoxide during efflux further increased channel activity, indicating that diazoxide can facilitate functional recovery of CBZ rescued channels. Clinically, this has important implications, as it has been proposed that small changes in K_ATP_ channel activity are correlated with large differences in clinical outcome (Macmullen et al., 2011). Interestingly, we have found that diazoxide precludes glibenclamide's ability to rescue K_ATP_ trafficking mutants, while it has no effect on the rescue of mutant channels by CBZ when the drugs are co-administered. This further supports the clinical feasibility of a combination therapy using a pharmacological chaperone and a channel activator to alleviate symptoms in patients with CHI. Also important, the effects of CBZ were observed in two physiologically relevant systems, namely the rat β-cell line INS-1 and freshly isolated primary human β-cells. Further, the concentrations at which CBZ is effective at rescuing TMD0 trafficking mutants (10–50 μM) is similar to those used to block Na^+^ channels, suggesting the approved dosage prescribed for CBZ will impact K_ATP_ channel expression. These data make a compelling case for further exploration of CBZ as a potential treatment for patients with certain forms of CHI.

Besides therapeutic implications, CBZ and glibenclamide differ markedly in their chemical structures and yet both rescue the expression of only those K_ATP_ channels with trafficking mutations in TMD0 of SUR1 and both inhibit K_ATP_ channel activity. An intriguing question to address in the future is whether the closed channel conformation rendered by these ligands represents a state that favors forward trafficking.

### The role of diazoxide on K_ATP_ channel trafficking

Ideally, a pharmacological chaperone would correct protein trafficking defects without compromising or even enhance protein function. In this regard, it is worth noting that the K_ATP_ channel opener diazoxide has been reported to correct channel trafficking defects caused by the SUR1 mutations R1394H (Partridge et al., 2001). Using a stable HEK293 cell line co-expressing wild-type or R1394H His-tagged hamster SUR1 and Kir6.2 tagged at the C-terminus with a HMA (heart muscle kinase phosphorylation site)-FLAG epitope, Partridge et al. showed that the mutant SUR1 failed to reach the cell surface and instead accumulated in the trans-Golgi network, and that diazoxide was able to restore surface expression of the R1394H mutant SUR1. However, a subsequent study by Yan et al. using FLAG-tagged R1394H mutant hamster SUR1 transiently co-expressed with Kir6.2 in COS cells reported normal trafficking of the mutant to the cell surface (Yan et al., 2004). Whether these different results are due to the different constructs or cells used remain to be resolved. Aside from the R1394H mutation reported by Partridge et al., no other known trafficking mutations tested so far are rescued by diazoxide (Yan et al., 2004, 2007; Chen et al., 2013b). Also worth noting, Powell et al. showed that a diazoxide analog BPDZ 154 restored ATP-inhibited channel activity in human β-cells from a CHI patient with the homozygous *ABCC8* intronic mutation c.1467+5G>A after 24–48 h incubation. Since it is not clear how this intronic mutation affects channel trafficking and/or function, it remains to be determined whether BPDZ 154 enhances channel trafficking and/or gating to restore function.

## Interplay between channel expression and gating in disease manifestation

Although some TMD0 mutations only impair channel trafficking such that pharmacological rescue of mutant channels to the cell surface is expected to partially or fully restore channel function, some impact both channel biogenesis and gating as exemplified by the R74W and E128K mutations (Pratt et al., 2009). These mutations result in CHI by preventing channel expression at the cell surface. Upon rescue to the cell surface by tolbutamide followed by washout, mutant channels exhibit reduced sensitivity to ATP inhibition, a gating defect commonly observed in gain-of-function mutations associated with permanent neonatal diabetes (PNDM). Detailed analysis revealed that these two mutations cause functional uncoupling between SUR1 and Kir6.2 by impairing the ability of SUR1 to hypersensitize Kir6.2 channels to ATP inhibition (Pratt et al., 2011). When these mutations were introduced into the insulinoma cells INS-1 and rescued to the cell surface by tolbutamide, subsequent washout of tolbutamide led to cells with more hyperpolarized membrane potential in the face of glucose stimulation (Pratt et al., 2009), similar to β-cells with gain-of-function, diabetes-causing mutant K_ATP_ channels.

Intuitively, trafficking defects are associated with loss of channel function and the disease CHI. However, it has been shown that many mutations identified in PNDM (Gloyn et al., 2004; Proks et al., 2004, 2005, 2006; Koster et al., 2005) also reduce channel biogenesis efficiency, including Q52R, V59G/M, R201C/H and I296L in Kir6.2 (Lin et al., 2006a) as well as F132L in SUR1 (Pratt et al., 2009) when expressed heterologously as homomeric mutant channels (Table 2). It is interesting that glibenclamide was also found to significantly improve surface expression of heterologously expressed homomeric mutant channels (Lin et al., 2006a), again hinting at SUR1-Kir6.2 interactions in sulfonylurea-mediated rescue. Whether CBZ also improves surface expression of these PNDM mutations remains to be determined. Because these PNDM mutations are dominant, heterozygous mutations with severe gating defects, a mutant channel subunit can exert its gain-of-function gating effect by co-assembly with the WT allele. In this scenario, the extent of expression of mutant subunit in the cell surface channel population may determine the extent of overall channel gating defect and thus, disease severity as has been proposed for several PNDM mutations, including V324M in SUR1 (Zhou et al., 2010) as well as C42R and Pro226-Pro232 deletion mutation in Kir6.2 (Yorifuji et al., 2005; Lin et al., 2013).

**Table 2:** **Neonatal Diabetes-associated K_ATP_ channel trafficking mutations and response to sulfonylurea treatment**.

**Mutation**	**Surface expression increased by SU**	**Gating property**	**References**
**SUR1**			
F132L	Yes	Increased P_o_	Pratt et al., 2009
V324M	N.D.	Increased MgADP sensitivity	Zhou et al., 2010
**Kir6.2**			
C42R	N.D.	Increased P_o_	Yorifuji et al., 2005
Q52R	Yes	Increased P_o_	Proks et al., 2004; Lin et al., 2006a
V59G	Yes	Increased P_o_	Proks et al., 2004; Lin et al., 2006a
V59M	Yes	Increased P_o_	Koster et al., 2005; Lin et al., 2006a
R201C	Yes	Decreased ATP inhibition	Proks et al., 2004; Lin et al., 2006a
R201H	Yes	Decreased ATP inhibition	Proks et al., 2004; Lin et al., 2006a
Pro226_Pro232del	N.D.	Increased P_o_	Lin et al., 2013
I296L	Yes	Increased P_o_	Proks et al., 2005; Lin et al., 2006a

The above studies highlight the importance of the interplay between channel expression and gating defects in determining disease phenotype. As K_ATP_ conductance is a product of the number of channels in the β-cell membrane and the open probability of the channel at a given metabolic state dictated by channel gating properties, correlation between channel defects and disease phenotype would require thorough analysis of the impact of a mutation on both channel expression and channel gating as well as consideration of the genetic context of the mutation.

## Conclusions and perspectives

Pharmacological chaperones have emerged as promising therapeutic tools for treating diseases resulting from defective protein folding and/or trafficking. Demonstration that sulfonylureas and CBZ are effective pharmacological agents able to restore surface expression of K_ATP_ trafficking mutants identified in congenital hyperinsulinism has direct clinical relevance. As CBZ is an FDA-approved drug, it may stand to rapidly improve current therapies for patients harboring trafficking mutations within TMD0 of SUR1. In order to spur translation of these findings into real treatment, an important next step is to demonstrate the efficacy of CBZ in β-cells isolated from CHI patients with K_ATP_ trafficking mutations within TMD0. Also, currently only a subset of identified TMD0 trafficking mutations associated with disease has been examined for their ability to be rescued by CBZ. The therapeutic applicability of CBZ for treating K_ATP_ trafficking disorders will likely expand in the future as more mutations are identified and tested. Finally, although CBZ also inhibits K_ATP_ channel function, this inhibition is reversible and can be partially overcome by co-application of potentiators, such as diazoxide, without compromising CBZ's corrector effect. These findings represent significant improvements over pharmacological rescue using glibenclamide, but CBZ itself may only be a model demonstrating the potential that future pharmacological correctors hold for treating K_ATP_ channel trafficking disorders.

Beyond the therapeutic implications, *in vitro* studies utilizing pharmacological chaperones and naturally occurring mutations in K_ATP_ channel subunits have enhanced our understanding of structure-function relationships in terms of biogenesis and molecular assembly, as well as gating and coupling between subunits. For instance, the finding that only trafficking mutations within TMD0 of SUR1 are amenable to pharmacological rescue further underscores the importance of this unique domain in mediating subunit interactions with Kir6.2 and highlights a role of TMD0 in channel assembly. The fact that drug binding, at least in the case of sulfonlylureas, to L0 and TMD2 of SUR1 as well as N-terminus of Kir6.2 has functional consequences for mutations within TMD0 suggests that either these domains can physically interact or there is a mechanism for transducing structural changes in trans to TMD0. Further, the fact that some K_ATP_ trafficking mutants rescued by reversible inhibitors (CBZ, tolbutamide) are functional upon drug washout and retain normal responses to metabolic signals and pharmacological ligands implies that these residues in TMD0 are not involved in gating or other functional aspects of the channel, but likely play important roles in the folding of this domain or may even be key residues at the interface of SUR1 and Kir6.2. At present, it is unclear whether CBZ binds directly to the channel complex during biogenesis or impacts channel expression and gating indirectly through interactions with other proteins. If CBZ does interact directly, defining the binding sites on K_ATP_ will provide valuable information on the mechanism by which this drug modulates channel folding, assembly and gating. This knowledge is critically important for future efforts to design more effective drugs that will target the biogenesis or gating defects of disease-causing mutant K_ATP_ channels.

### Conflict of interest statement

The authors declare that the research was conducted in the absence of any commercial or financial relationships that could be construed as a potential conflict of interest.

## Abbreviations

CBZ: carbamazepine
CF: cystic fibrosis
CFTR: cystic fibrosis transmembrane conductance regulator
CHI: congenital hyperinsulinism
K_ATP_: ATP-sensitive potassium channel
Kir6.2: inwardly rectifying potassium channel 6.2
NBD: nucleotide binding domain
PNDM: permanent neonatal diabetes
SU: sulfonylureas
SUR1: sulfonylurea receptor 1
TMD: transmembrane domain.
